# The Synthesis of Zinc Complex of Salicylaldehyde Serine Schiff Base and Assessment of Its Efficiency as a Heat Stabilizer for Poly (Vinyl Chloride)

**DOI:** 10.3390/polym17233119

**Published:** 2025-11-24

**Authors:** Feng Ye, Zhihao Yan, Haoran Ma, Kangjie Guo, Cheng You, Qingsong Zheng, Shafeng Lv, Xiaodong Wang, Qiufeng Ye, Yeqian Ge, Zhuanyong Zou, Chi Shen

**Affiliations:** 1 College of Textile Science and Engineering, Shaoxing University, Shaoxing 312000, Chinazouzhy@usx.edu.cn (Z.Z.); 2 Zhejiang Province Key Laboratory of Clean Dyeing and Finishing Technology, Shaoxing 312000, China; 3 Zhejiang Wafa Ecosystem Science & Technology Co., Ltd., Shaoxing 312452, China; 4 Shaoxing Testing Institute of Quality and Technical Supervision, Shaoxing 312000, China

**Keywords:** polyvinyl chloride, serine, Schiff base, heat stabilizer, synergistic effect

## Abstract

The zinc complex of salicylaldehyde serine Schiff base (ZnL) was synthesized from serine, salicylaldehyde, and zinc diacetate and subsequently applied as a heat stabilizer in poly (vinyl chloride) (PVC). The structure of ZnL was determined using elemental analysis, crucible thermogravimetric method, infrared spectroscopy, thermogravimetric analysis and ^1^H NMR spectra. The heat stability effect of ZnL for PVC was investigated using the Congo red and oven aging methods. The results indicated that PVC stabilized by ZnL exhibited a certain degree of original whiteness and long-term heat resistance. In contrast with PVC stabilized by ZnSt_2_ and Ca/Zn, ZnL was found to be slightly inferior in terms of whiteness but superior in long-term heat resistance. It was observed that complexation of ZnL with CaSt_2_ could enhance both the original whiteness and long-term heat resistance of PVC, while also alleviating the “zinc burning” phenomenon. In contrast, complexation with ZnSt_2_ was found to promote “zinc burning” for PVC. Furthermore, the heat stability mechanism of ZnL for PVC was explored through experiments focusing on HCl absorption and active chlorine substitution. The results demonstrated that ZnL could replace active chlorine on the PVC molecule and absorb HCl gas. Finally, auxiliary heat stabilizers such as pentaerythritol (Pe), dibenzoyl methane (DBM), and epoxidized soybean oil (ESBO) were added to ZnL/CaSt_2_ to evaluate their synergistic effects. It was found that ESBO in PVC exhibited the best synergistic effect with ZnL/CaSt_2_ and was superior to those observed with DBM and Pe. When the ratio of ZnL/CaSt_2_/ESBO was set at 0.6/2.4/0.9, PVC demonstrated the optimal thermal stability performance.

## 1. Introduction

Poly (vinyl chloride) (PVC), as an important thermoplastic polymer, is produced through the free radical polymerization of vinyl chloride (VC) monomers [1,2]. Owing to its cheap price, exceptional chemical stability, good biocompatibility, and outstanding mechanical properties, this material is widely used in numerous industrial fields, including chemical production, construction engineering, packaging materials, and electronic component manufacturing [3,4,5]. With the continuous increase in society’s demand for environmentally friendly materials and multifunctional properties, PVC formulations that combine eco-friendly characteristics, low toxicity, high transparency, and antibacterial functions have received growing attention. This trend is driving the development of PVC stabilizers toward multifunctional integration [6,7,8].

From the perspective of molecular structure, structural defects in the main chain of PVC, such as unstable structures in allylic chloride and tertiary chloride, significantly reduce its thermal stability. When the material is exposed to high-temperature processing environments, it is prone to dehydrochlorination reactions, forming conjugated diene structures, which in turn lead to product discoloration (yellowing to browning) and deterioration of mechanical properties (reduced impact strength and increased brittleness) [9,10]. To conquer this technical confine, it is required to introduce highly efficient thermal stabilizing systems during PVC processing, which can improve processing stability and ensure long-term aging resistance through mechanisms such as interrupting chain degradation reactions chemically, chelating metal impurities, and replacing unstable chlorine atoms. Currently, the development of multifunctional composite thermal stabilizers has become an important research direction in this field [11,12,13,14].

The current PVC heat stabilizer market is dominated by lead salts, organotins, and metal soaps. Among them, lead salt stabilizers have been banned by environmental regulations in many countries due to their lead content, which is neurotoxic and easily causes environmental pollution [15]. Although organotin stabilizers have remarkable thermal stability, their application has been greatly limited owing to the high price and the biotoxicity of certain types [16]. Calcium-zinc compound stabilizers in metal soaps have become the mainstream choice for industrial applications at present due to their advantages of being free from heavy metal pollution and having a low cost [17]. In recent years, organic nitrogen-based heat stabilizers, which are based on the electronic structure of nitrogen atoms, have developed rapidly [18,19,20]. The unique lone pair electrons of nitrogen give it strong coordination ability, allowing it to efficiently capture the hydrogen chloride generated during PVC thermal degradation and form stable complexes. Based on this characteristic, researchers have combined organic nitrogen-containing groups with calcium and zinc metal soaps through molecular design and have successfully developed organic nitrogen-based heat stabilizers, which have both high thermal stability and environmental adaptability [21,22]. This new system continues the environmental benefits of calcium-zinc stabilizers while significantly improving thermal stability efficiency through nitrogen-metal synergistic effects, demonstrating important application prospects and innovative potential [23,24].

Recently, an increasing number of researchers have begun to focus on organic nitrogen-based heat stabilizers and have found that these stabilizers exhibit excellent performance. Zhimin Fu and colleagues synthesized a Schiff base heat stabilizer called Zn-HBAPA. The result suggested that Zn-HBAPA could exhibit excellent thermal stability for flexible PVC, compared to the cadmium-zinc soap stabilizers used in the industry. And it could be found that the system in which CaSt_2_ and tris(hydroxymethyl) aminomethane(THAM) were mixed with it in different ratios could enhance the thermal stability of PVC while maintaining its original whiteness [25]. Zhixuan Cui and colleagues synthesized a zinc Schiff base heat stabilizer called BSE-Zn using salicylaldehyde and ethylenediamine as raw materials. The results suggested that the PVC samples stabilized by BSE-Zn could exhibit great thermal stability, and their thermal stabilization time could reach up to 90 min, proving that it was a long-term heat stabilizer. The thermal stability of PVC was improved by blending CaSt_2_ with BSE-Zn, and the original whiteness was also enhanced. When the ratio of CaSt_2_/BSE-Zn was set at 2:1, the PVC achieved the optimal thermal stability [26]. Subsequently, Nan Wang and colleagues condensed histidine with vanillin to synthesize a Schiff base called VanHis, and then reacted VanHis with anhydrous zinc acetate to prepare VanHis-Zn. Results showed that VanHis-Zn could delay “zinc burning”. Compared with commercially available calcium/zinc stearate stabilizers, VanHis-Zn could have good thermal stability. When mixed with different proportions of Ca(acac)_2_, the original whiteness and long-term heat resistance of PVC samples could be significantly improved [27].

Serine is a natural amino acid that mainly participates in protein synthesis, neurotransmitter metabolism, and immune function regulation, with effects such as promoting brain health, enhancing immunity, and improving skin condition. In this paper, a zinc complex of ZnL was synthesized using serine, salicylaldehyde, and zinc dihydrate as raw materials. The heat stability effect of ZnL for PVC was then studied, and the mechanism of ZnL for PVC was further discussed. In addition, the thermal stability effects of ZnL in combination with CaSt_2_ or ZnSt_2_ were studied, and based on ZnL/CaSt_2_, the synergistic thermal stability effects of three auxiliary thermal stabilizers (Pe, DBM, ESBO) were explored.

## 2. Experiment

### 2.1. Materials

PVC resin was bought from Xinjiang Tianye Group Co. LTD (Xinjiang, China). Calcium carbonate was bought from Zhejiang Himpton New Materials Co. LTD (Zhejiang, China). Calcium stearate (CaSt_2_), zinc stearate (ZnSt_2_), dioctyl phthalate (DOP, 98%), salicylaldehyde, serine acid (99%), zinc acetate dihydrate, pentaerythritol (Pe), dibenzoylmethane (DBM), epoxidized soybean oil (ESBO), were bought from Aladdin Reagent Company. And the thermal stabilizer Ca/Zn was made from CaSt_2_ and ZnSt_2_ with a mixing ratio of approximately 1:1.

### 2.2. Synthesis and Characterization of ZnL

**Figure 1:**
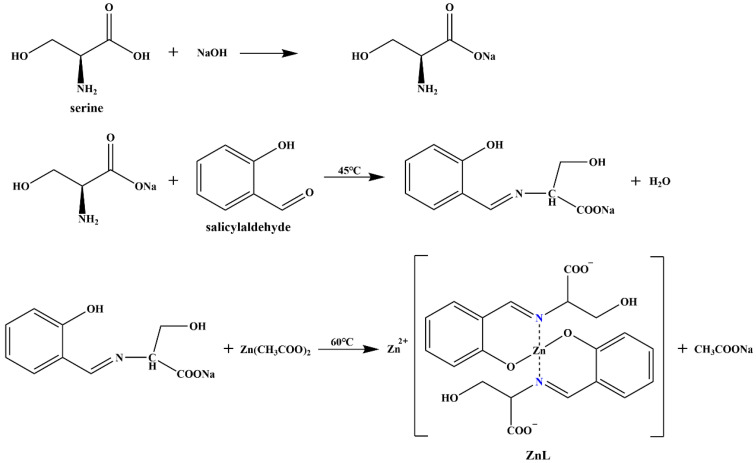
Synthesis of ZnL.

Figure 1 showed the schematic diagram of the synthesis process of ZnL.The sodium hydroxide (0.80 g, 20 mmol) was first dissolved in 50 mL of pure water to prepare a sodium hydroxide solution. Subsequently, the serine (2.102 g, 20 mmol) and salicylaldehyde (2.442 g, 20 mmol) were separately added. The mixed solution was transferred to a 150 mL conical flask containing a magnetic stir bar after thorough mixing. The conical flask was sealed, and the solution was uniformly stirred in a 45 °C water bath for 2 h. In another clean beaker, the zinc acetate dihydrate (4.39 g, 20 mmol) was dissolved in 50 mL of pure water to prepare the zinc acetate solution. The conical flask was then transferred to a 60 °C water bath, and the zinc acetate solution was slowly added. After the dropwise addition was finished, the mixture in the conical flask was stirred for about 30 min. The hot filtration was carried out after the reaction ended, and the filter cake was washed with pure water until the pH of the filtrate reached neutrality. The filter cake was transferred to an oven at 70 °C and dried for 5 h to obtain the expected pale yellow product, which was the zinc complex of ZnL.

The structure of ZnL was characterized using the following experimental methods [28]. The zinc content in ZnL was determined using the crucible thermogravimetric method, which involved roasting at 700 °C for 3 h in a vacuum tube furnace. The contents of carbon, hydrogen, nitrogen, and other elements in ZnL were ascertained by an elemental analyzer (Euro EA 3000, EA Instruments, Milan, Italy). The molecular structure of ZnL was confirmed using infrared spectroscopy (IR Prestige21, Shimadzu Corporation, Japan) in the range of 4000–400 cm^−1^. The thermogravimetric curve of ZnL was measured by a thermogravimetric analyzer (TG, TG/DTA 6300, Japan) with a heating rate of about 10 °C/min from room temperature to 800 °C in an air atmosphere. The BRUKER AVANCE III HD 400 MHz (AVANCE NEO, Bruck, Switzerland) was used to record the data of ^1^H nuclear magnetic resonance (NMR) of ZnL at room temperature. In this process, DMSO-d_6_ was used as a solvent.

### 2.3. Preparation of PVC Film

PVC powder resin (12 g), PVC paste resin (12 g), CaCO_3_ (3.6 g), and heat stabilizer (0.72 g) were weighed by an electronic balance, combined in a mortar, and finely ground. The mixture was transferred to a disposable plastic cup, followed by the dropwise addition of DOP (12 g) with stirring until uniform [29]. It was then poured into a glass mold, clamped, and placed in an oven at 140 °C for 30 min for plasticization and molding. The resulting PVC film was finally removed and stored.

### 2.4. Evaluation of Thermal Stability Performance

#### 2.4.1. Static Congo Red Method

Following the uniform grinding of PVC powder resin (5 g) with heat stabilizer (0.15 g) in a mortar, the mixture was transferred into a glass test tube using a square of filter paper. A Congo red test paper, held by a rubber-stoppered capillary tube, was inserted such that its end was approximately 20 mm above the powder surface. The test tube was then clamped and immersed in a 180 °C oil bath, ensuring the oil level exceeded the height of the powder. The stabilization time was recorded as the period required for the test paper to turn blue completely [30,31].

#### 2.4.2. Oven Discoloration Method

Rectangular samples (15.0 × 20.0 × 1.0 mm) were cut from the prepared PVC film and placed horizontally on an aluminum plate. The assembly was heated in an oven at 180 °C [32]. At 10 min intervals, a sample was removed and mounted on a blank sheet of paper. This process was repeated until all samples had turned black. Finally, the paper containing the samples was scanned to create a tabular image for assessing the whiteness change.

#### 2.4.3. Research on the Mechanism of ZnL Stabilizing PVC

To further validate the mechanism of ZnL on PVC thermal stabilization, this article conducted two verification experiments as follows [33].

The first experiment aimed to verify the HCl absorption capability of ZnL. An appropriate amount of ZnL was placed in a three-necked flask and subjected to a flow of dry HCl gas at 180 °C for 1 h, followed by dry air at 140 °C for 2 h to remove residual HCl. A control group without the introduction of dry HCl gas was set up, and dry air was continuously introduced throughout the experiment. The reaction products from both experiments were washed, filtered, and the filtrates were tested with 0.1 M AgNO_3_ for white precipitate formation. The residues were dried and analyzed by IR spectroscopy for comparison.

The second experiment was performed to determine if ZnL could replace the active chlorine in PVC. A mixture of PVC powder resin and ZnL was ground in a mortar, transferred to a glass bottle, and heated at 180 °C for 50 min. A control experiment used pure PVC powder under identical conditions. After heating, the products from both groups were washed and filtered. The filtrates were tested with 0.1 M AgNO_3_ for the formation of a white precipitate, while the solid residues were dried and analyzed by IR spectroscopy, with their spectra compared to those of pristine ZnL and the unheated PVC/ZnL mixture.

## 3. Results and Discussion

### 3.1. Characterization of ZnL

The crucible heat weight test revealed that the mass of zinc oxide at the end of calcination accounted for 27.83% of the mass of ZnL before calcination, and the calculated zinc content in ZnL is 22.33%. The analysis results of ZnL elements were listed in Table 1. Based on the ratio of the mass percentages of zinc and nitrogen atoms, it was determined that the ratio of zinc and nitrogen atoms in the molecular formula was 1:1. Under the simple assumption regarding the content of crystalline water, some possible theoretical values of material elements were listed. Consequently, the molecular formula of ZnL was deduced to be Zn_2_(C_10_H_9_NO_4_)_2_·5/2H_2_O.

**Figure 2:**
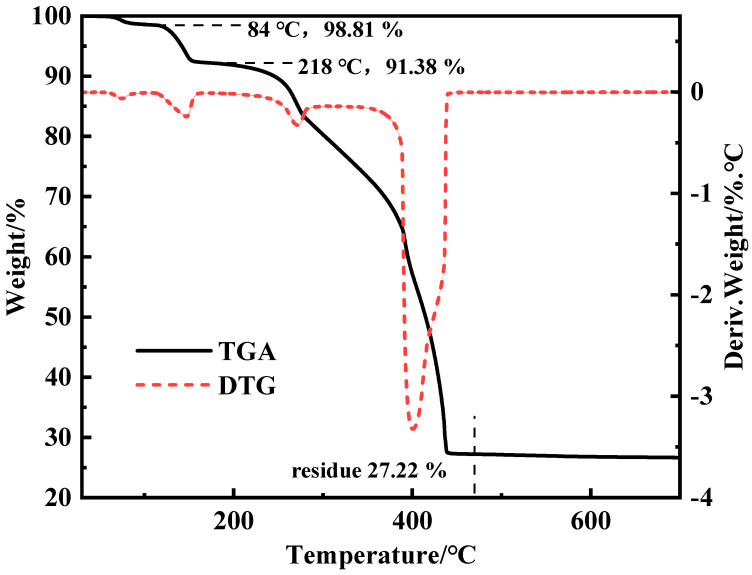
TGA and DTA curves of ZnL.

The thermogravimetric curve of ZnL was presented in Figure 2 using a thermogravimetric analyzer [34]. Based on the curve of ZnL, a mass loss of 1.19% at 84 °C was observed, which was due to the removal of free water from ZnL. A further mass loss of 7.43% was recorded for the sample at 218 °C, which was caused by the removal of crystalline water from ZnL. Meanwhile, the theoretical mass loss value for ZnL containing 5/2 crystalline water was calculated to be 7.64%, which was found to be very close to the actual value observed for ZnL. This indirectly verified the result of the above-mentioned elemental analysis involving 5/2 crystalline water. The molding temperature of PVC typically ranges from 160 to 220 °C, and it was found that the mass fraction of ZnL remained at 90% even at 245 °C. This indicated that ZnL was stable at the processing temperatures of PVC and met the basic requirements for being a PVC heat stabilizer. The mass fraction of ZnL stabilized at 27.22% and no longer decreased after heating at 540 °C, indicating that the thermal degradation of ZnL had been completed. The mass fraction of ZnL obtained from this result was basically consistent with that obtained from the crucible TG experiment (27.83%). It could, thus, be proven again that the molecular formula of the synthesized ZnL was Zn_2_(C_10_H_9_NO_4_)_2_·5/2H_2_O.

**Figure 3:**
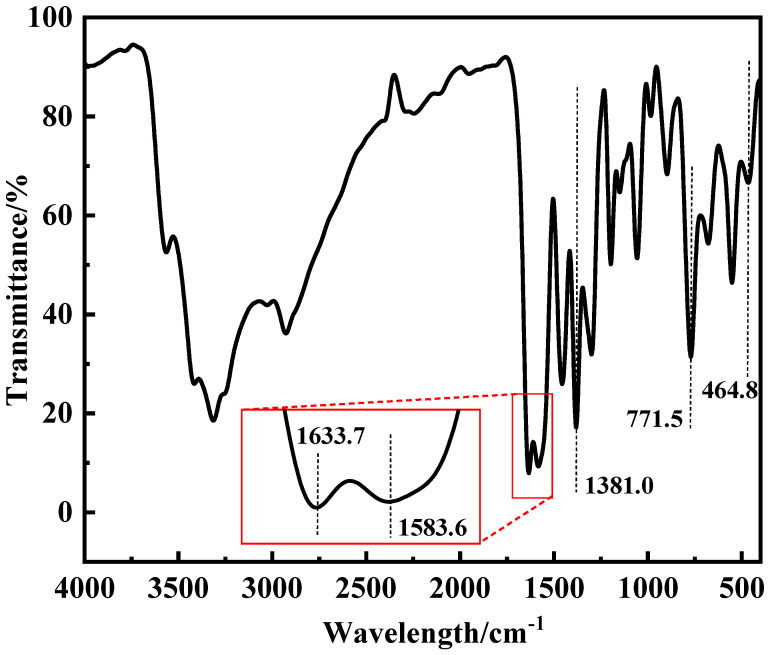
Infrared spectrum of ZnL.

Figure 3 showed the infrared absorption spectra of ZnL, wherein certain characteristic absorption peaks provide important information regarding the structure of ZnL [35]. In the figure, an absorption peak observed at 1583.6 cm^−1^ was identified as the stretching vibration peak of the imine structure (C=N). Additionally, two absorption peaks, located at 1633.7 cm^−1^ and 1381.0 cm^−1^, were, respectively, due to the asymmetric and symmetrical stretching vibration peaks of the carboxylate structure (-COO-). At the same time, the peak detected at 771.5 cm^−1^ was regarded as the characteristic peak of the Zn-N structure. Its presence indicated the coordination between nitrogen and zinc atoms within the imine structure (C=N). Furthermore, after consulting relevant data, it was discovered that the peak at 464.8 cm^−1^ corresponded to the typical peak of the Zn-O bond. This finding suggested that oxygen atoms on the phenol group had combined with zinc atoms to form Zn-O bonds. When combined with the results from the aforementioned TGA and EA, the existence of these characteristic peaks confirmed that the ZnL had been successfully synthesized.

**Figure 4:**
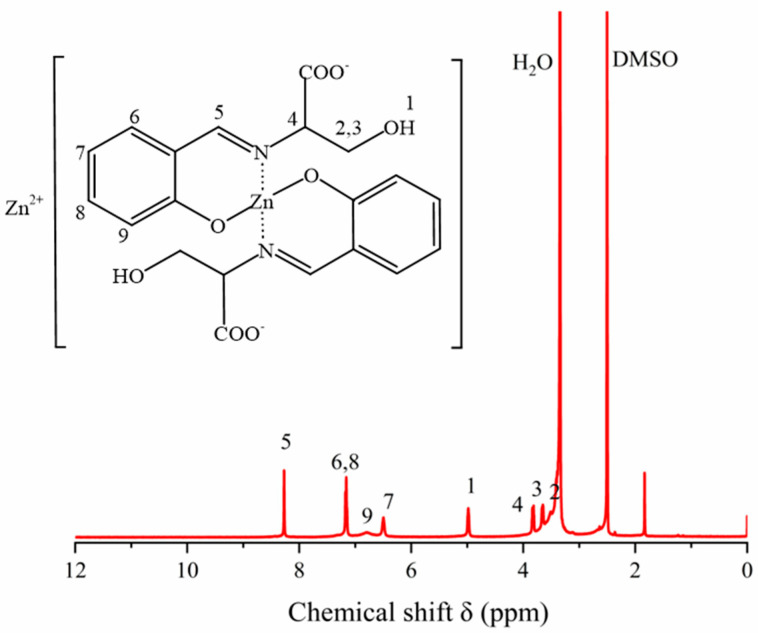
^1^H NMR spectra of ZnL.

The ^1^H NMR spectrum of ZnL was shown in Figure 4. The ^1^H NMR spectrum of ZnL was recorded in DMSO-d_6_. The chemical shift δ = 2.51 corresponded to the characteristic peak of the solvent DMSO-d_6_, and the chemical shift δ = 3.34 corresponded to the characteristic peak of the H_2_O. From the ^1^H NMR spectrum of ZnL, the chemical shifts at 6.0~7.5 ppm (m, 6–9) corresponded to the four hydrogens on the ortho-substituted phenyl groups; the chemical shift δ = 8.27 (s, 5) corresponded to one hydrogen of the carbon-nitrogen double bond (HC=N); the shifts at 3.0~4.0 ppm (m, 2–4) corresponded to three hydrogens on the methylene (CH_2_) and methine (CH) groups; the chemical shift δ = 4.98 (s, 1) corresponded to 1 hydrogen of the hydroxyl group (OH). The above confirmed the synthesis of the final product ZnL.

### 3.2. The Heat Stability Effect of ZnL for PVC

#### 3.2.1. Static Congo Red Test

**Figure 5:**
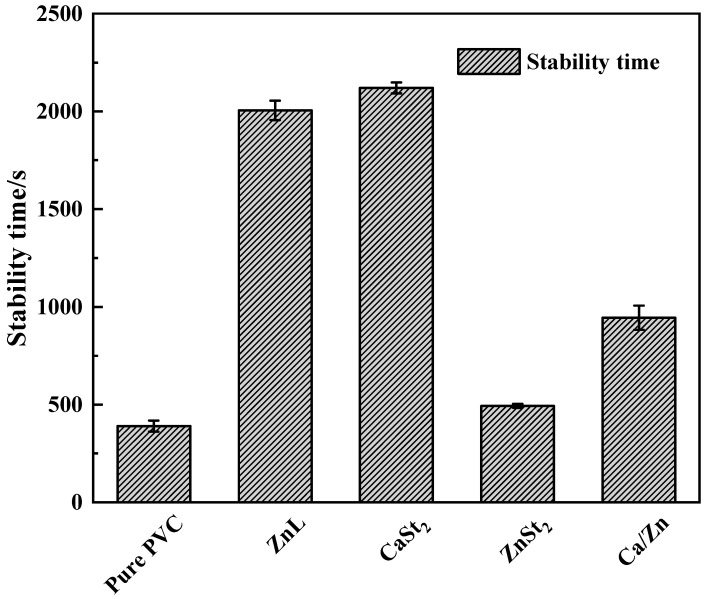
Static Congo red time of PVC stabilized by different thermal stabilizers.

Results of the Static Congo red test on PVC stabilized with several heat stabilizers were displayed in Figure 5. As could be observed from the figure, the static Congo red time of PVC had been improved by the addition of several heat stabilizers [36]. The static Congo red time achieved with ZnL was found to be close to that of CaSt_2_, indicating good long-term heat resistance. This finding suggested that ZnL also exhibited superior HCl absorption capabilities and was more effective in restraining thermal decomposition of PVC.

#### 3.2.2. Oven Discoloration Test

**Figure 6:**
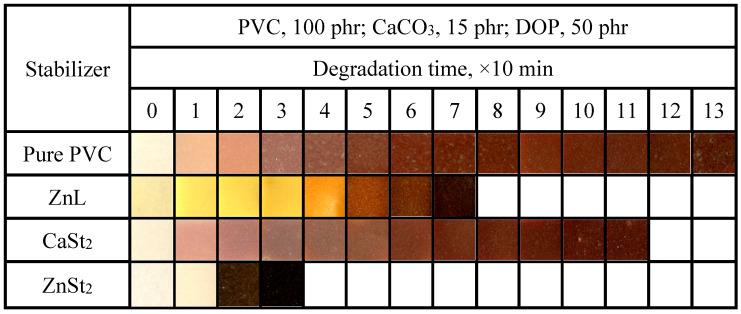
Discoloration performance of PVC samples stabilized by different thermal stabilizers at 180 °C.

The aging conditions of PVC films stabilized with various heat stabilizers at 180 °C were shown in Figure 6. As shown in the figure, regarding long-term heat resistance, ZnL was found to be inferior to CaSt_2_ but superior to ZnSt_2_, which was similar to the results of the previously mentioned Congo red method. In addition to pure PVC and CaSt_2_-stabilized PVC, the other two types of heat-stabilized PVC samples all exhibited a certain degree of original whiteness. Among these, ZnSt_2_-stabilized PVC demonstrated the best original whiteness but was also the most severely affected by “zinc burning”, as its film rapidly turned black at an early stage, lacking long-term heat resistance [37]. PVC stabilized by ZnL displayed a certain level of original whiteness, indicating that ZnL had a certain substitution effect on the active chlorine in the PVC structure. On the other hand, the increased content of Schiff base structure in ZnL was found to be beneficial for improving the original whiteness.

### 3.3. Thermal Stabilization Mechanism of PVC by ZnL

**Figure 7:**
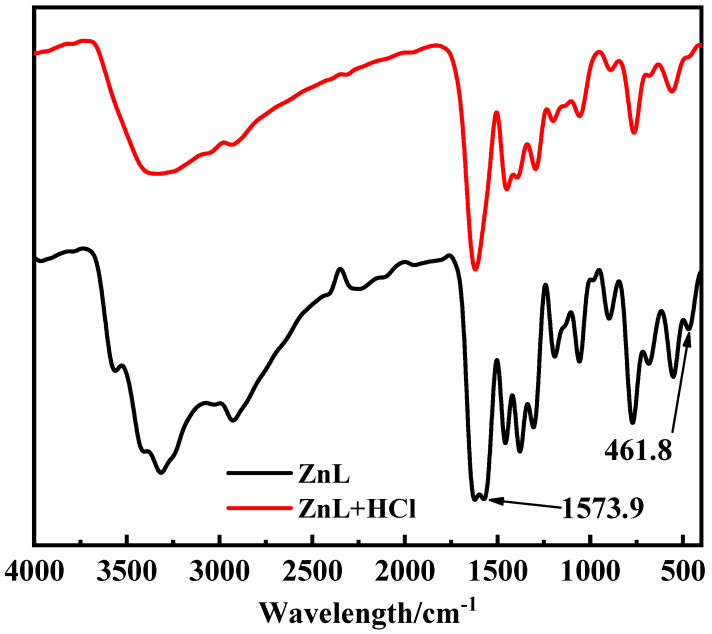
Infrared spectrum of ZnL with and without HCl treatment.

As seen from the aforementioned experiments, PVC stabilized by ZnL exhibited a certain level of original whiteness and good long-term heat resistance. To further study the mechanism of PVC stabilization by ZnL, two experiments were designed and conducted in this study. The experimental results were as follows:

In the test conducted to verify whether ZnL could absorb HCl gas, it was observed that no white precipitate formed after adding AgNO_3_ into the filtrate of the control group, which had not been exposed to HCl gas. In contrast, a distinct white precipitate was detected in the filtrate of the experimental group, which had been exposed to HCl gas. This indicated that chloride ions were present in the filtrate of the experimental group, suggesting that ZnL had reacted with HCl. The Infrared spectrum of ZnL, both with and without HCl treatment, was shown in Figure 7. It was found that the peaks of HCl-treated ZnL at 1573.9 cm^−1^ and 461.8 cm^−1^ disappeared. The peak at 1573.9 cm^−1^, identified as the typical peak of the imine structure, demonstrated that the imine group might play a role in the absorption of HCl. Meanwhile, the peak at 461.8 cm^−1^, belonging to the representative peak of the Zn-O structure, suggested that the Zn-O bond in ZnL might have reacted with HCl.

**Figure 8:**
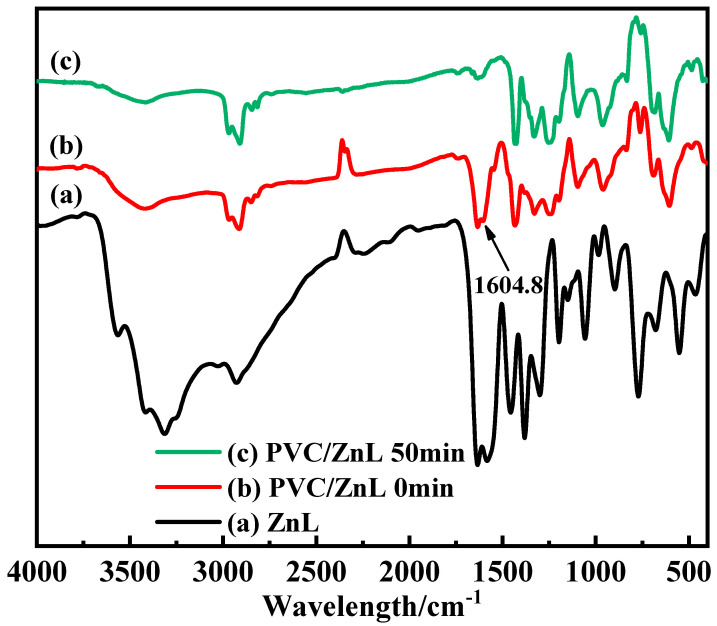
Some IR spectra of (a) ZnL, (b) PVC/ZnL, (c) PVC/ZnL after heating for 50 min.

In the experiment conducted to verify whether ZnL could replace active chlorine on the PVC structure, the white precipitate was observed in the filtrate of the experimental group (PVC/ZnL) after AgNO_3_ was added. In contrast, no white precipitate was detected in the filtrate of the control group (pure PVC) after the addition of AgNO_3_, indicating that chloride ions were present in the filtrate of the experimental group. These results demonstrated that ZnL had reacted with the active chlorine on the PVC structure. Additionally, Figure 8 showed the FTIR of ZnL, PVC/ZnL, and heated PVC/ZnL. It was noted that the peak located at 1604.8 cm^−1^ in curve (b), representing PVC/ZnL, disappeared in curve (c) after heating treatment. This absorption peak was identified as the characteristic peak of the imine structure (C=N). Combined with the detection of chloride ions in the filtrate of the previous experimental group (PVC/ZnL), it was suggested that the imine structure (C=N) might have reacted with the active chlorine in the PVC structure and could inhibit the thermal decomposition of PVC.

**Figure 9:**
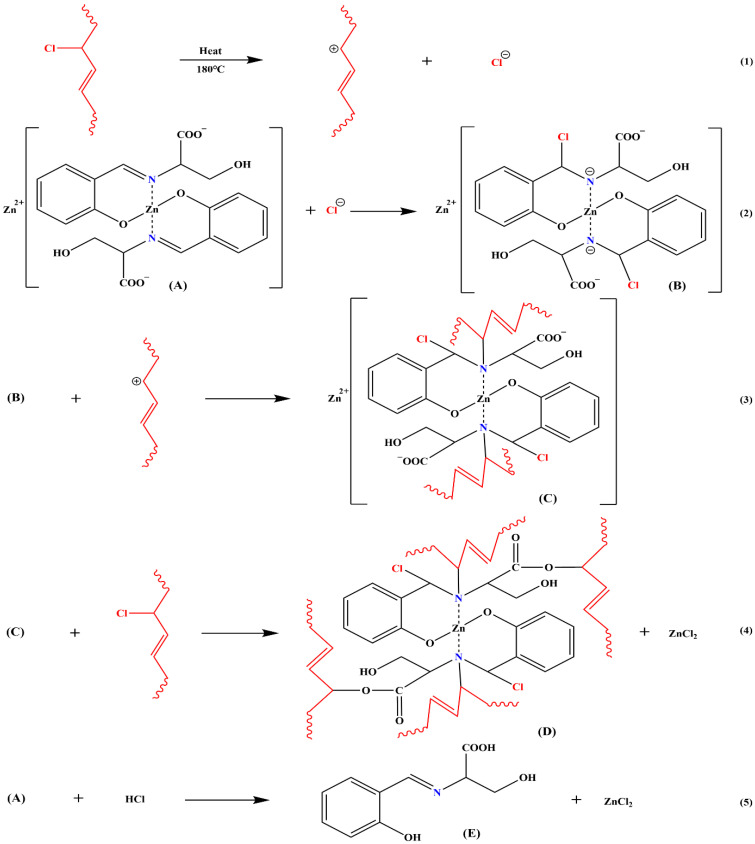
Mechanism of thermal stabilization of PVC by ZnL.

As shown in Figure 9, the heat stability mechanism of ZnL for PVC was derived. Active chlorine was heated and separated from the PVC chain [Equation (1)]. Free chloride ions were then observed to react with imines in ZnL, causing the destruction of the C=N and the formation of a C-Cl bond along with negatively charged nitrogen ions [Equation (2)]. These nitrogen ions were found to bind to previously dissociated PVC chain segments [Equation (3)]. The fatty acid salt-like structures present on ZnL were also capable of replacing active chlorine on the PVC chains [Equation (4)]. Additionally, the carboxylate structure of ZnL was demonstrated to have an absorption effect on HCl [Equation (5)]. In conclusion, ZnL was shown to delay the thermal degradation of PVC at its source by replacing active chlorine and could absorb HCl to prevent further degradation of PVC. ZnL was identified as a promising new heat stabilizer that required further development and refinement [38].

### 3.4. Compounding Study of ZnL with Other Thermal Stabilizers

Based on the aforementioned testing experiments of ZnL on PVC, it was observed that PVC stabilized by ZnL exhibited good long-term stability and a certain degree of initial whiteness. However, for its application in the actual production process of PVC, its comprehensive thermal stability effect needed to be further improved [39,40]. To obtain a more efficient thermal stabilizer, the compounding of ZnL was studied in this section. CaSt_2_, ZnSt_2_, Pe, DBM, and ESBO were selected to compound with ZnL, and the synergistic thermal stability effects among them were investigated.

**Figure 10:**
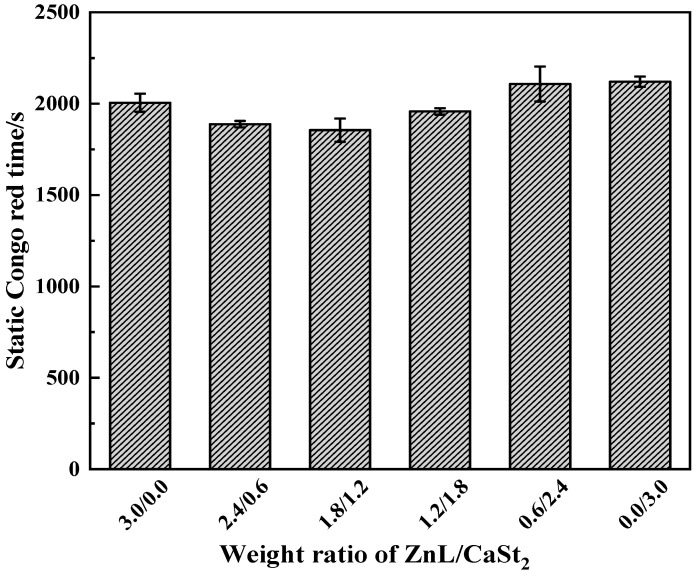
Static Congo red time of ZnL/CaSt_2_ stabilized PVC.

Figure 10 showed the Congo red test results for PVC stabilized by ZnL/CaSt_2_. As shown in the figure, the static Congo red times of PVC with different ZnL/CaSt_2_ ratios showed little variation and remained basically at the same level. This result might be due to the following reason: the static stability times of ZnL and CaSt_2_ were not significantly different when used individually, and no synergistic effect on HCl absorption was observed when ZnL and CaSt_2_ were used together. Therefore, ZnL and CaSt_2_ had little impact on the static Congo red time.

**Figure 11:**
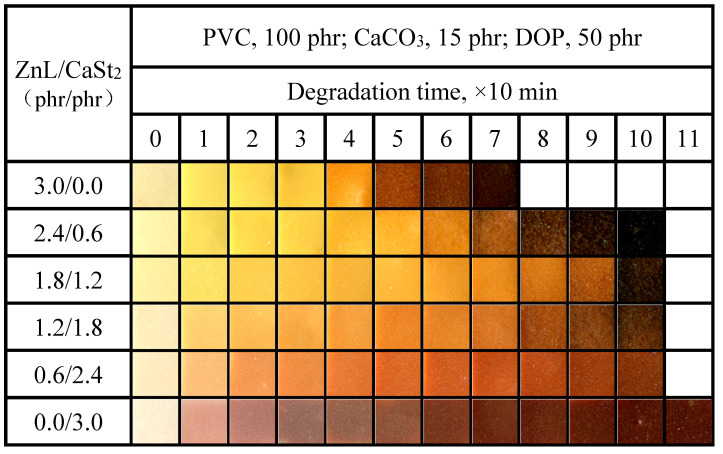
Discoloration performance of PVC samples stabilized with different ZnL/CaSt_2_ ratios at 180 °C.

Figure 11 showed the color-changing properties of PVC films stabilized by the combined use of ZnL and CaSt_2_. It was observed from the figure that, compared with ZnL alone, the time required for complete blackening of the film was delayed after the combination of ZnL and CaSt_2_, and the effect of “zinc burning” was further reduced. Compared with pure CaSt_2_, the original whiteness of the PVC film was strengthened by the combination of ZnL and CaSt_2_, indicating that a better synergistic effect had been achieved through their combination. The possible synergistic mechanism was that while ZnL took the place of the active chlorine on PVC, ZnCl_2_ was formed, which was not conducive to the thermal stability of PVC. The presence of CaSt_2_ in an appropriate amount was found to reduce the concentration of ZnCl_2_ and delay the “zinc burning”. Additionally, when the ratio of ZnL/CaSt_2_ was set at 1.8/1.2, the PVC exhibited the best original whiteness and good long-term heat resistance.

**Figure 12:**
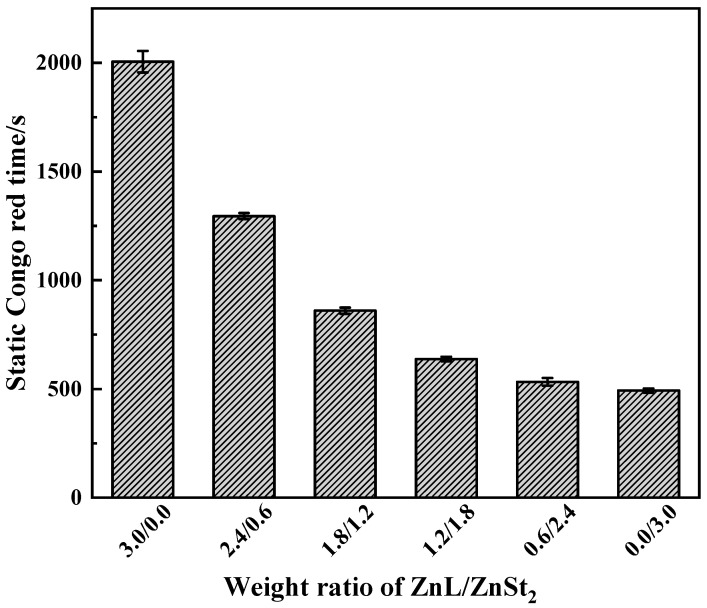
Static Congo red time of ZnL/ZnSt_2_ stabilized PVC.

Results of Congo red test on PVC stabilized by ZnL/ZnSt_2_ were shown in Figure 12. Obviously, due to the poor HCl absorption capacity of ZnSt_2_ itself, the higher the content of ZnSt_2_ in the composite heat stabilizer, the worse the Congo red standing time performance of PVC, indicating that the composite thermal stabilizer had a weak ability to absorb HCl, and also reflecting their lack of synergistic effect in HCl absorption.

**Figure 13:**
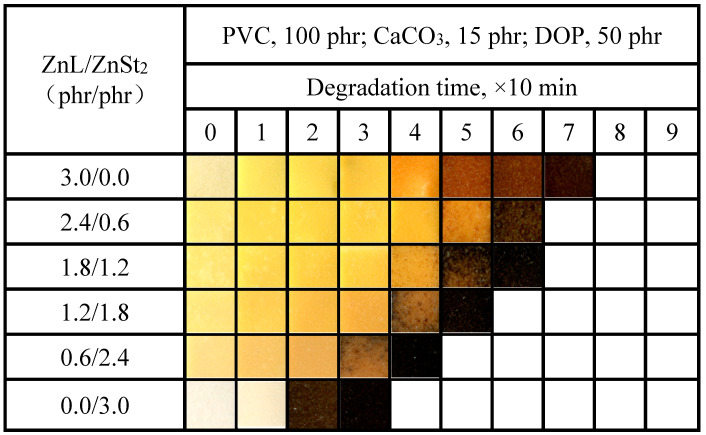
Discoloration performance of PVC samples stabilized with different ZnL/ZnSt_2_ ratios at 180 °C.

The color-changing properties of PVC films stabilized with the ZnL/ZnSt_2_ complex were shown in Figure 13. As shown in the figure, ZnSt_2_ could not help improve the original whiteness or reduce the yellowing of PVC stabilized by ZnL. On the contrary, the higher the ZnSt_2_ content in the composite heat stabilizer, the more pronounced the long-term heat resistance decline of the PVC film, and the earlier and more severely the effect of “zinc burning” occurred. This performance was also consistent with the previous test result by the Congo red method.

The above experiments indicated that the combined effect of ZnL and ZnSt_2_ was not ideal, and the overall thermal stability actually decreased. When ZnL was combined with CaSt_2_, both the initial whiteness and long-term heat resistance were improved [41,42]. Therefore, subsequent experiments will adopt a combination of ZnL/CaSt_2_ with three auxiliary stabilizers (Pe, DBM, ESBO) for joint formulation.

**Figure 14:**
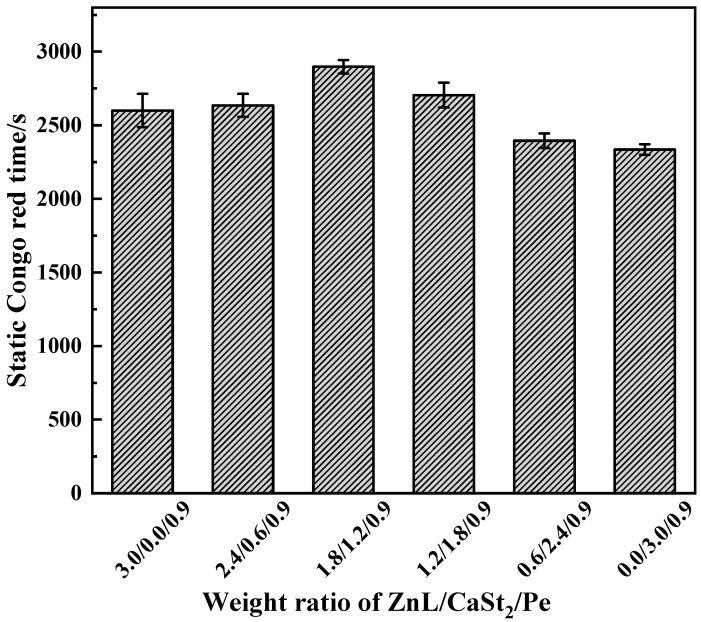
Static Congo red time of ZnL/CaSt_2_/Pe stabilized PVC.

Figure 14 showed the results of the Congo red test on PVC stabilized by ZnL/CaSt_2_/Pe. Compared with the results of ZnL/CaSt_2_ in Figure 10, it was found that Pe significantly increased the Congo red time of PVC stabilized by ZnL/CaSt_2_ at different ratios, indicating that Pe enhanced the ability to absorb HCl of the ZnL/CaSt_2_ thermal stabilization system. Simultaneously, along with the rise in CaSt_2_ content, the static Congo red time of PVC stabilized by ZnL/CaSt_2_/Pe showed a trend of first increasing and then decreasing. The static Congo red time of PVC was optimal when the ratio of ZnL/CaSt_2_/Pe was 1.8/1.2/0.9, up to 2898 s. This suggested that there was a synergy among ZnL, CaSt_2_, and Pe, and the combined thermal stabilizer of ZnL, CaSt_2,_ and Pe could better absorb HCl, thus extending the static Congo red time of PVC.

**Figure 15:**
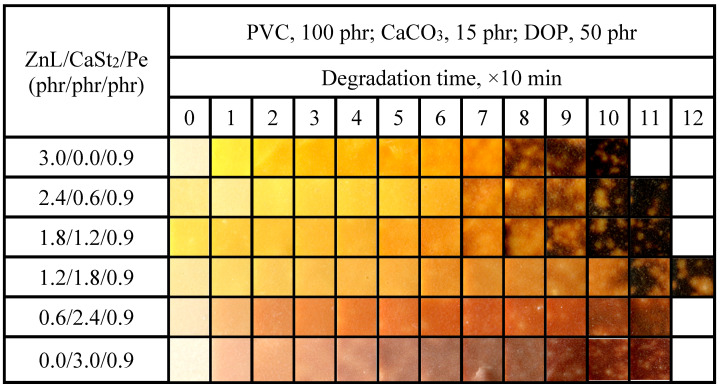
Discoloration performance of PVC samples stabilized with different ZnL/CaSt2/Pe ratios at 180 °C.

Figure 15 showed the color-changing properties of PVC films stabilized with different ZnL/CaSt_2_/Pe ratios stable at 180 °C. By comparing the oven discoloration of ZnL/CaSt_2_ in Figure 11, Pe could enhance the original whiteness and long-term heat resistance of PVC films. The long-term heat resistance of PVC films stabilized by ZnL/CaSt_2_/Pe was enhanced with the rise of CaSt_2_ content. When the ZnL/CaSt_2_/Pe ratio was 1.2/1.8/0.9, the PVC film sample only showed partial darkening of some areas after being heated to 180 °C for 100 min, and it had still not completely blackened after 120 min. At this ratio, PVC exhibited the best overall thermal stability, meaning it had the optimal original whiteness and good long-term heat resistance. These research results indicated that ZnL, CaSt_2_, and Pe had a synergistic effect on the substitution of active chlorine on PVC and the absorption of HCl gas, thereby effectively strengthening the overall thermal stability of PVC.

According to the above analysis, the synergistic stability mechanism of ZnL, CaSt_2_ and Pe could be concluded as follows: in the structure of ZnL, the fatty acid salt structure and the imine group (C=N) in the Schiff base structure could take place of the unstable chlorine on PVC, and the imine group (C=N) would produce the Lewis acid (ZnCl_2_) during the substitution process, which would cause the “zinc burning” and accelerate the thermal degradation of polyvinyl chloride. However, CaSt_2_ and Pe could react with ZnCl_2_ to form more stable complexes, CaCl_2_ and Pe-Cl, reducing the concentration of ZnCl_2_ to mitigate “zinc burning”. At the same time, CaSt_2_ also generated ZnSt_2_ during the reaction, which contributed to the original whiteness of PVC. In addition, ZnL, CaSt_2_, and Pe could all absorb HCl gas, ensuring the long-term heat resistance of PVC [43,44]. Through the synergistic action of the above aspects, the combined formulation of ZnL, CaSt_2_, and Pe could effectively improve the overall stability of PVC.

**Figure 16:**
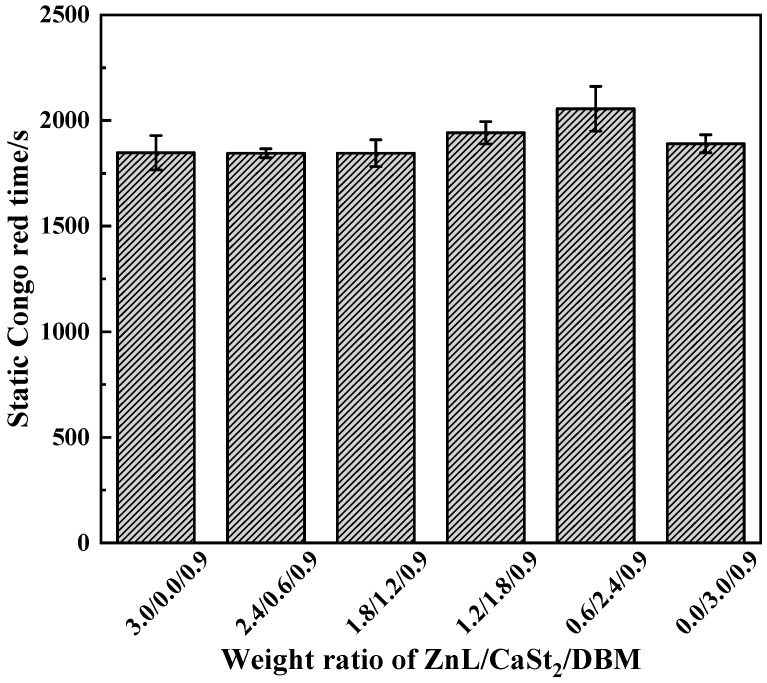
Static Congo red time of ZnL/CaSt_2_/DBM stabilized PVC.

Experimental results measured by the Congo Red method of PVC stabilized with ZnL/CaSt_2_/DBM were shown in Figure 16. It could be seen that the static Congo red time of PVC stabilized in different proportions still remained at the same level. Compared with the results in Figure 10, this level line even decreased slightly. This indicated that DBM could not enhance the HCl absorption ability of PVC stabilized by ZnL/CaSt_2_. On the contrary, DBM weakened it, which was unfavorable for the long-term heat resistance of PVC.

**Figure 17:**
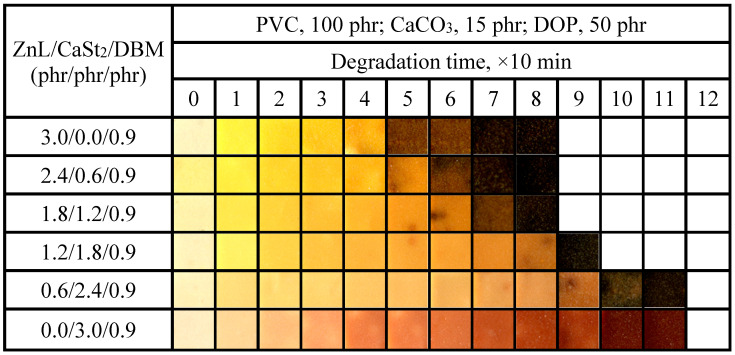
Discoloration performance of PVC samples stabilized with different ZnL/CaSt_2_/DBM ratios at 180 °C.

The aging condition of PVC films stabilized with ZnL/CaSt_2_/DBM was shown in Figure 17. It could be observed that when the content of CaSt_2_ was relatively high, the long-term heat resistance of PVC would be better. By comparing the oven discoloration results of ZnL/CaSt_2_ without DBM in Figure 11, the addition of DBM caused the PVC film samples to turn black earlier, which was unfavorable for the long-term heat resistance of PVC films. This result was consistent with the previous experiment result of Congo red. However, it could also be found that DBM enhanced the original whiteness of PVC film stabilized by ZnL/CaSt_2_, although DBM was still insufficient in improving the yellowish tint of it. When the ZnL/CaSt_2_/DBM ratio was 0.6/2.4/0.9, PVC exhibited the best overall thermal stability, with both original whiteness and long-term heat resistance being relatively good. According to the results above, it was found that DBM could enhance the original whiteness of PVC stabilized by ZnL/CaSt_2_, but it reduced its long-term heat resistance.

**Figure 18:**
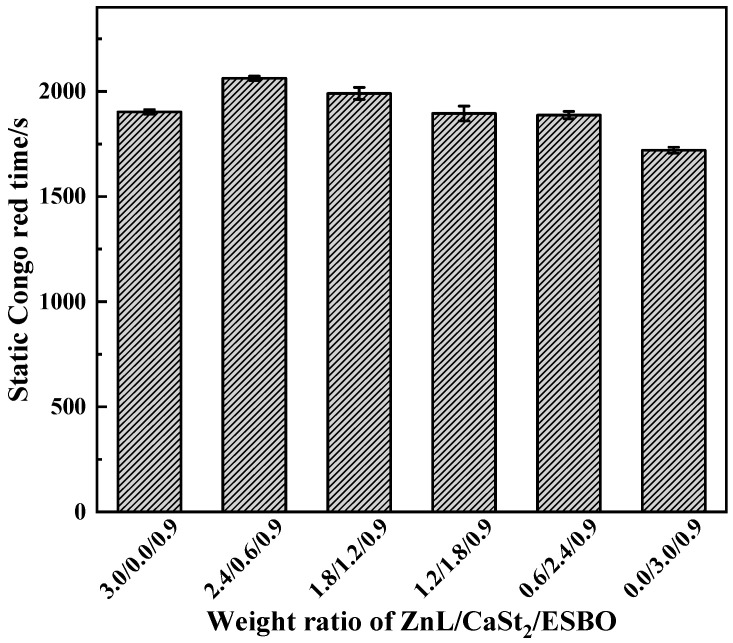
Static Congo red time of ZnL/CaSt_2_/ESBO stabilized PVC.

Figure 18 showed the Congo red time of PVC stabilized with ZnL/CaSt_2_/ESBO. It could be observed that along with the rise of CaSt_2_ content, the static Congo red time of PVC showed a trend of first increasing and then decreasing. By comparing the static Congo red time of ZnL/CaSt_2_ in Figure 10, it was found that ESBO could not significantly enhance the static Congo red time of PVC stabilized by ZnL/CaSt_2_.

**Figure 19:**
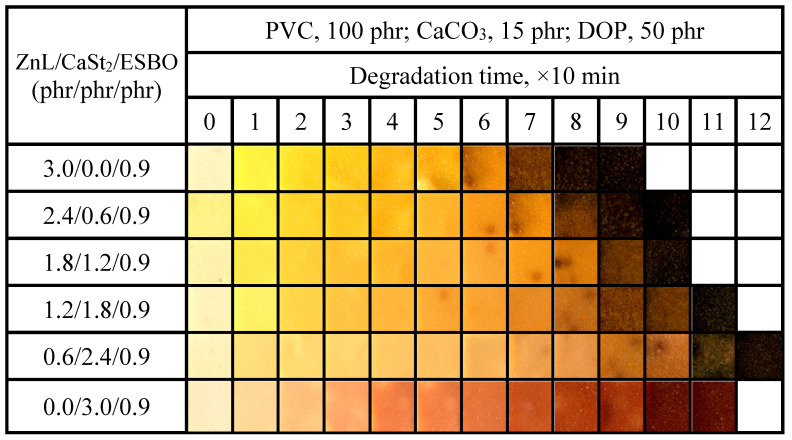
Discoloration performance of PVC samples stabilized with different ZnL/CaSt_2_/ESBO ratios at 180 °C.

Figure 19 showed the color-changing properties of PVC films stabilized with ZnL/CaSt_2_/ESBO. It could be observed that the higher the content of CaSt_2_ in the compound heat stabilizer, the better the long-term heat resistance of PVC film. In comparison with the results in Figure 11, it was found that the addition of ESBO had a slight enhancement on the original whiteness and long-term heat resistance of PVC, but the yellowish color of it was not completely improved [45,46]. When the ratio of ZnL/CaSt_2_/ESBO was 0.6/2.4/0.9, PVC had the best original whiteness and long-term heat resistance, indicating that the combination of ZnL, CaSt_2,_ and ESBO had a certain synergy on the thermal stability of PVC. Based on the results above, ESBO could enhance the overall thermal stability of PVC stabilized by ZnL/CaSt_2_, and the increase rate was greater than that of Pe.

## 4. Conclusions

In this paper, a zinc complex of ZnL was synthesized by the precipitation method using serine and salicylaldehyde as raw materials. The chemical element composition and content of ZnL were confirmed by means of elemental analysis, thermogravimetric analysis, and the crucible thermogravimetric method. The molecular formula was estimated to be Zn_2_(C_10_H_9_NO_4_)_2_·5/2H_2_O. Then, the heat stability effect of ZnL for PVC was characterized by the Congo red method and the oven discoloration method. It was found that PVC stabilized by ZnL had certain original whiteness and long-term heat resistance. In terms of long-term heat resistance, ZnL was comparable to CaSt_2_, but the whiteness was weaker than that of ZnSt_2_ and Ca/Zn, and the color of the PVC film was generally yellowish, which needed to be further improved. In addition, the ability of ZnL to take the place of active chlorine on PVC and absorb HCl gas was experimentally verified, and its possible mechanism for stabilizing PVC was explained.

ZnL was compounded separately with CaSt_2_ and ZnSt_2_. Results from the static Congo red method and oven discoloration method indicated that complexation of ZnL with CaSt_2_ could enhance the original whiteness and long-term heat resistance of PVC, and effectively mitigate the “zinc burning”, whereas complexation of ZnL with ZnSt_2_ could promote the occurrence of “zinc burning”. Therefore, subsequent experiments studied the combined thermal stabilization effects of ZnL/CaSt_2_ with three auxiliary thermal stabilizers (Pe, DBM, ESBO). The results showed that PVC stabilized synergistically by ZnL/CaSt_2_/Pe could improve both original whiteness and long-term heat resistance; DBM enhanced the original whiteness of PVC stabilized by ZnL/CaSt_2_, but somewhat reduced its long-term heat resistance; the addition of ESBO improved both the original whiteness and long-term heat resistance of the ZnL/CaSt_2_ thermal stability system, with a greater improvement than Pe. This paper provides a new approach for the development of a new type of composite heat stabilizer.

## Figures and Tables

**Table 1:** Elemental analysis results of ZnL.

Formula	Value of Calculation (%)			
	Zn	C	H	N
ZnL	23.90	44.12	3.31	5.15
Zn_2_(C_10_H_9_NO_4_)_2_·H_2_O	23.13	42.70	3.56	4.98
Zn_2_(C_10_H_9_NO_4_)_2_·2H_2_O	22.41	41.38	3.79	4.83
**Zn_2_(C_10_H_9_NO_4_)_2_·5/2H_2_O**	**22.07**	**40.75**	**3.90**	**4.75**
Zn_2_(C_10_H_9_NO_4_)_2_·3H_2_O	21.74	40.13	4.01	4.68
Zn_2_(C_10_H_9_NO_4_)_2_·4H_2_O	21.10	38.96	4.22	4.55
	Experimental value (%)			
**Reality**	**22.33**	**39.78**	**4.18**	**4.51**

## Data Availability

The data in the main text is exactly the data we need; no new data has been created.
